# 

**Published:** 1872-06

**Authors:** 


Contents of June Number.

### ORIGINAL DEPARTMENT.

ART. I.—Albuminuria in Pregnancy and its relation to puerperal Convulsions. By Geo. T. Campbell, M. D., Skaneateles, N. Y. . . . . . . . 405
ART. II.—On the recent Advances in the Theory of Vision. By H. Helmholtz. Translated by F. W. Abbott, M. D. . . . . . . . . . . . . . . . 416
ART. III.—Report of a case of Aneurismal Tumor following penetrating wound of shoulder. Operation for ligation or right Subsclavian Artery. By C. C. F. Gay, M. D. . . . . . . . . . . . . . . . . . 424

### MISCELLANEOUS.

Two cases illustrating the production of Vowel and Consonant Sounds. By Prof. E. M. Moore, M. D., Rochester, N. Y. . . . . . . . . . . 428

Darwinism in Reference to the Eyes of Animals. . . . . . . . . . . . . 434

Removal of a Large Fibro-Cystic Tumor of the Uterus by Gastrotomy. By Prof. T. Gaillard Thomas, M. D., New York. . . . . . . . . . 436

### EDITORIAL DEPARTMENT.

OVARIOTOMY.—Looking after our Laurels. . . . . . . . . . . . . . . . 437
Niagara County Medical Society, Old and New. . . . . . . . . . . . . . 438

BOOKS REVIEWED:

Lectures on Aural Catarrh; or the commonest forms of Deafness and their cure. By Peter Allen, M. D. . . . . . . . . . 438

A Treatise of Diseases of the Bones. By Thomas M. Markoe, M. D. . . . . . . . . . . . . . . . . . . . 439

Earth as a Topical application in Surgery. By Addinell Hewson, M. D. . . . . . . . . . . . . . . . . 439

History of Medicine from the earliest ages to the commencement of the Nineteenth Century. By Robley Dunglison, M. D., LL. D. Edited by Richard J. Dunglison, M. D. 440

Dr. Rigbys' Obstetric Memoranda. By Alfred Meadows, M. D. . . . . . . . . . . . . . . . . . . . 440

Memoranda on Poisons. By the late Thomas Hawkes Tanner, M. D., F. L. S. . . . . . . . . . . . . . . 440

Parturition without Pain. By M. L. Holbrook, M. D. . . . . . . 441

Diseases of Women. By T. Gaillard Thomas M. D. . . . . . . 441

NEW BOOKS.—The Periodicals . . . . . . . . . . . . . . . . . 442

THE LENS.—The Popular Science Monthly.—The Globe Microscope. . . . . . . . . . . . . . . . . . . . 443

Western Virginia Medical Society. . . . . . . . . . . . . . . 443

Mortality of the U. S. . . . . . . . . . . . . . . . . . . 444

Books and Pamphlets Received. . . . . . . . . . . . . . . 444

